# Flubendazole demonstrates valid antitumor effects by inhibiting STAT3 and activating autophagy

**DOI:** 10.1186/s13046-019-1303-z

**Published:** 2019-07-08

**Authors:** Shichong Lin, Lehe Yang, Yulei Yao, Lingyuan Xu, Youqun Xiang, Haiyang Zhao, Liangxing Wang, Zhigui Zuo, Xiaoying Huang, Chengguang Zhao

**Affiliations:** 1 School of Pharmaceutical Sciences, Wenzhou Medical University, Wenzhou, Zhejiang, 325035 People’s Republic of China; 2 The First Affiliated Hospital, Wenzhou Medical University, Wenzhou, Zhejiang, 325000 People’s Republic of China; 3 The Institute of Life Sciences, Wenzhou University, Wenzhou, Zhejiang, 325035 People’s Republic of China

**Keywords:** Flubendazole, Colorectal cancer, STAT3, Autophagy, Apoptosis

## Abstract

**Background:**

Signal transducer and activator of transcription 3 (STAT3) is an oncogene, which upregulates in approximately 70% of human cancers. Autophagy is an evolutionarily conserved process which maintains cellular homeostasis and eliminates damaged cellular components. Moreover, the STAT3 signaling pathway, which may be triggered by cancer cells, has been implicated in the autophagic process.

**Methods:**

In this study, we found that the anthelmintic flubendazole exerts potent antitumor activity in three human colorectal cancer (CRC) cell lines and in the nude mouse model. The inhibition of cell proliferation in vitro by flubendazole was evaluated using a clonogenic assay and the MTT assay*.* Western blot analysis, flow cytometry analysis, siRNA growth experiment and cytoplasmic and nuclear protein extraction were used to investigate the mechanisms of inhibiting STAT3 signaling and activation of autophagy induced by flubendazole. Additionally, the expression of STAT3 and mTOR was analyzed in paired colorectal cancer and normal tissues collected from clinical patients.

**Results:**

Flubendazole blocked the IL6-induced nuclear translocation of STAT3, which led to inhibition of the transcription of STAT3 target genes, such as *MCL1*, *VEGF* and *BIRC5*. In addition, flubendazole also reduced the expression of P-mTOR, P62, BCL2, and upregulated Beclin1 and LC3-I/II, which are major autophagy-related genes. These processes induced potent cell apoptosis in CRC cells. In addition, flubendazole displayed a synergistic effect with the chemotherapeutic agent 5-fluorouracil in the treatment of CRC.

**Conclusions:**

Taken together, these results indicate that flubendazole exerts antitumor activities by blocking STAT3 signaling and inevitably affects the autophagy pathway. Flubendazole maybe a novel anticancer drug and offers a distinctive therapeutic strategy in neoadjuvant chemotherapy of CRC.

**Electronic supplementary material:**

The online version of this article (10.1186/s13046-019-1303-z) contains supplementary material, which is available to authorized users.

## Background

Colorectal cancer (CRC) is one of the most common digestive tract malignancies worldwide, which is among the leading causes of cancer death. Meanwhile, the high incidence rate of CRC maintains a significant increase [1, 2]. Despite the many advances in CRC research, including screening and treatment, the overall survival rate of patients with CRC is still low and the rate of tumor recurrence remains currently invariable [3]. Therefore, researchers have intense interest in finding new strategies aimed at improving treatment effectiveness and prognosis [4].

The oncogenic transcription factor signal transducer and activator of transcription 3 (STAT3) is overactive in most of human cancers including CRC [5]. Compelling evidence has demonstrated the crucial role of STAT3 in promoting tumor cell proliferation, angiogenesis, metastasis and resistance to therapies by regulating the expression of correlative genes such as vascular endothelial growth factor (VEGF) [6–8]. In addition, STAT3 is associated with tumor cell apoptosis through the regulation of BCL2, BAX, MCL1 and survivin (*BIRC5*) [9–11]. Hence, inhibiting the STAT3 signaling axis has gradually emerged as an important strategy for the treatment of cancer [12].

Autophagy, a form of programmed cell death, is involved in cellular growth, autoimmunity and tumor progression as a conserved intracellular degradation system [13]. Increasing evidence demonstrates that numerous therapeutic strategies induce cell apoptosis through upregulation of autophagy [14]. However, the specific molecular mechanisms connecting apoptosis and autophagy are still not deciphered. Thus, the study of autophagy activation should provide new insights into the antitumor effect mechanism mediated through autophagy [15].

Flubendazole is a well-known anthelmintic drug that is widely used to treat infections of worm and intestinal parasites in clinical practice. The anthelmintic action of flubendazole is based on altering microtubule structure, inhibition of tubulin polymerization and disruption of microtubule function [16]. Notably, other research groups have suggested that flubendazole is a potential antitumor agent [17]. However, although several studies have reported various mechanisms and pathways mediating the antitumor effect of flubendazole, the precise mechanism remains unclear [18, 19]. In this study, we confirmed that flubendazole effectively inhibits cell proliferation and induces apoptosis in human CRC by blocking the STAT3 signaling axis and activation of autophagy. Meanwhile, flubendazole demonstrated synergistic effect with 5-Fluorouracil in human CRC cells. These findings suggest that flubendazole may be a novel antitumor therapeutic candidate drug in CRC treatment strategy in clinical practice.

## Materials and methods

### Antibodies and reagents

Flubendazole and 5-Fluorouracil were purchased from Sigma-Aldrich (St. Louis, MO, USA). All the cell culture reagents were purchased from Invitrogen Life Technologies (Carlsbad, CA, USA). Antibodies of GAPDH(#5174), STAT3 (#12640), Survivin (#2808), MCL1 (#94296), P-mTOR (#5536), mTOR (#2983), Beclin 1 (#3495) and LC3-I/II (#4108) were purchased from Cell Signaling Technology (Danvers, MA, USA). Antibodies of P-STAT3 (ab76315), VEGF (ab52917), Lamin B1 (ab16048), BAX (ab32503) and Ki67(ab156956) were purchased from Abcam Co. (Cambridge, MA, USA). Antibody of BCL2 (sc-7382) was purchased from Santa Cruz Biotechnology Inc. (Dallas, TX, USA). The horseradish peroxidase (HRP)-conjugated donkey anti-rabbit IgG and HRP-conjugated goat anti-mouse IgG were purchased from Santa Cruz Biotechnology Inc. (Dallas, TX, USA). Total Protein Extraction Kit was purchased from Boster Biological Technology, (Wuhan, China). The siRNAs against STAT3 (si-STAT3), corresponding negative control siRNA (si-NC) were designed and synthesized by GenePharma (Shanghai, China). Lipofectamine 3000 Transfection Kit was purchased from Invitrogen Life Technologies (Carlsbad, CA, USA). The Caspase3 Colorimetric Assay Kit was purchased from Abcam Co. (Cambridge, MA, USA). FITC Annexin V Apoptosis Detection Kit I and Propidium Iodide (PI) were purchased from BD Pharmingen (Franklin Lakes, NJ). TUNEL Apoptosis Assay Kit was purchased from Beyotime Institute of Biotechnology (China).

### Cell lines and culture

The human colorectal cancer cell lines HCT116, RKO, SW480, the normal human liver cell LO2 and cardiomyocytes H9C2 were purchased from Cell Resources Center of the Shanghai Institutes for Biological Sciences (Chinese Academy of Sciences, Shanghai, China). The short tandem repeat (STR) DNA profiles for HCT116, SW480 and RKO are shown in Additional files 2, 3 and 4. HCT116 cell line was maintained in Mc Coy’s 5A medium supplemented with 10% fetal bovine serum (FBS). RKO and SW480 cell lines were cultured in Roswell Park Memorial Institute (RPMI) 1640 medium supplemented with 10% FBS. Human liver cell LO2 was cultured in RPMI 1640 medium supplemented with 15% FBS. Human normal cardiomyocytes H9C2 was cultured in Dulbecco’s Modified Eagle Medium (DMEM) supplemented with 10% FBS. Routinely, cells were incubated at 37 °C in an atmosphere of 5% CO_2_.

### Methyl thiazolyl tetrazolium (MTT) assay

The colorectal cancer cells and normal human cells were seeded into 96-well plate at a density of 3000–5000 cells per well in different medium. The cells were incubated at 37 °C in 5% CO_2_. Test compounds (flubendazole or 5-fluorouracil) were dissolved in DMSO. Cells were maintained with test compounds for 48 h before the MTT assay. The plate was then incubated in a CO_2_ incubator for 4 h, crystals dissolved with 150 μL DMSO, and analyzed in a Microplate Reader at 490 nm. The half maximal inhibitory concentrations (IC_50_) value was calculated by Graphpad Prism 7.0 software.

### Clonogenic assay

Cell clonogenic assay was conducted as previously described [20]. Cancer cells were seeded into 6-well plate (500 cells per well). After cancer cell attachment, different concentrations of test compounds were added and incubated for 24 h. After 7 days, colonies were fixed by 4% paraformaldehyde, washed with phosphate-buffered saline (PBS) and stained with 0.1% crystal violet at room temperature.

### Western blot analysis

Tissue and cell protein were extracted using tissue or cell protein lysate buffer (Total Protein Extraction Kit). Cancer cells were seeded in 6-well plate and treated with different concentrations of flubendazole for 24 h, after that lysed with ice-cold lysate buffer. Proteins were separated by 10% or 12% sodium dodecyl sulfate-polyacrylamidegel (SDS-PAGE). And then proteins were transferred onto a polyvinylidene fluoride (PVDF) membrane and blocked with 5% skim milk for 1.5 h. The blots were incubated with specific primary antibody in Tris-Buffered Saline and Tween 20 (TBST) overnight at 4 °C. Then, the blots were incubated for 1 h at room temperature with horseradish peroxidase-conjugated secondary antibodies and washed three times with 1 × TBST. The density of the immunoreactive bands was analyzed using Image J computer software.

### Transient transfection of small interfering RNA (siRNA)

The siRNAs against STAT3 (si-STAT3), corresponding negative control siRNA (si-NC) were designed and synthesized by GenePharma (Shanghai, China). The process of transient cell transfection was conducted standardly according to manufacturers’ instructions. Sequences of siRNAs were shown as follows: STAT3 siRNA STAT3-Homo-398 5′-CCACUUUGGUGUUUCAUAATT-3′; STAT3 siRNA STAT3-Homo-978 5′-GCAACAGAUUGCCUGCAUUTT-3′; STAT3 siRNA STAT3-Homo-1070 5′-CCCGUCAACAAAUUAAGAATT-3′.

### Cytoplasmic and nuclear protein extraction

Cytoplasmic and nuclear proteins of HCT116 cells were detached through the NE-PER Nuclear &Cytoplasmic extraction kit (Thermo, Waltham, MA, USA). HCT116 cells were treated with flubendazole for 24 h, stimulating with IL6 for 30 min before been lysed. Cells were lysed using nuclear and cytoplasmic protein extraction Kit according to the manufacturer’s protocol. Protein expression of the cytoplasmic and nuclear extractions was determined by immunoblot analysis respectively.

### Flowcytometry

HCT116, RKO and SW480 cells were treated with different concentrations of flubendazole for approximately 48 h. Apoptotic cells were measured by flow cytometry using Apoptosis Detection Kit I. All samples were analyzed on a flowcytometer (BD Biosciences) and data was evaluated using FlowJo software.

### Determination of caspase 3 activity

Caspase3 activity of cell lysates was measured by a caspase 3 assay kit (Abcam, Cambridge, MA, USA) according to the manufacturer’s protocol. 1–5 × 10 ^6^ CRC cells were collected after treatment with different concentrations of flubendazole (0, 0.3, 0.6 or 1.2 μM) about 24–48 h. Activity of caspase3 was normalized by the protein concentration of the corresponding cell lysate. The activity was measured at 405 nm through SpectraMAX iD3 (Molecular Devices, San Jose, CA, USA).

### Hoechst 33342 staining

HCT116 and RKO cells were seeded in 6-well cell culture plate and treated with different concentrations of flubendazole for 12 h. Following fixation with 4% paraformaldelyde for 15 min at room temperature, cells were washed by PBS and stained with Hoechst 33342 for 20 min. Finally, cells were observed by fluorescence microscope (Nikon, Tokyo, Japan) using appropriate filters for blue fluorescence.

### Animal model

All animal care and experimental studies were performed according to the guidelines and approval of the Wenzhou Medical College Animal Policy and Welfare Committee. Female BALB/c athymic nude mice (6–8 weeks) were bred and maintained at the animal experimental center in Wenzhou Medical University. For colorectal cancer xenograft model, HCT116 cancer cells were harvested and subcutaneously implanted (5 × 10 ^6^ cells in 100 μL of PBS) into the right flank of mice. Once tumor volumes reached ~ 100 mm^3^, mice were divided into three different groups which were no obvious differences in mean body weights or tumor volumes. The treatment groups (6 mice per group) were treated with flubendazole 10 mg/kg or 30 mg/kg by intraperitoneal (i.p.) injection every other day. The tumor volumes were measured length (l), width (w) and calculating volume (V = 0.5 × l × w^2^) before every injection. On day 14 and 2 h after the last treatment of flubendazole, all mice were executed. The tumors were removed and prepared for western blot analysis. Tumor weight was measured. Hearts, livers, kidneys and lungs were fixed immediately and paraffin-embedded. The sections were subjected to H&E staining.

### Immunohistochemistry (IHC) analysis

Tumor tissue sections (4 μm) were deparaffinized, rehydrated and incubated with primarily Ki67 and P-STAT3 antibodies. HRP-conjugated secondary antibodies were used for detection. Images were obtained with Leica microscope.

### Apoptosis assay (TUNEL staining**)**

A One Step TUNEL Apoptosis Assay Kit (Beyotime, China) was used to detect apoptosis of tumor tissues sections (4 μm). The process of Tunel apoptosis assay was conducted standardly according to manufacturers’ instructions. Cell nuclei was stained with 4′,6-diamidino-2-phenylindole (DAPI), and fluorescence was evaluated by fluorescence microscopy.

### Patient samples

This study was approved by the Institutional Research Human Ethical Committee of the Wenzhou Medical University for the use of clinical biopsy specimens and informed consent was obtained from the patients. A total of 12 CRC patients biopsy samples were obtained. Clinical diagnosis was performed at the First Affiliated Hospital of Wenzhou Medical University. CRC tissues and matched tumor-adjacent morphologically normal CRC tissues were frozen and stored in liquid nitrogen until further analyses.

### Statistical analysis

All experiments were assayed at least three times except animal models. All statistical analyses were performed using GraphPad Prism 7.0 (GraphPad Software, CA, USA). Data are expressed as mean ± Standard deviation (SD). Students t-test was used to compare two groups (*P*-value < 0.05 was considered statistically significant).

## Results

### Flubendazole inhibits CRC cells proliferation

**Fig. 1:**
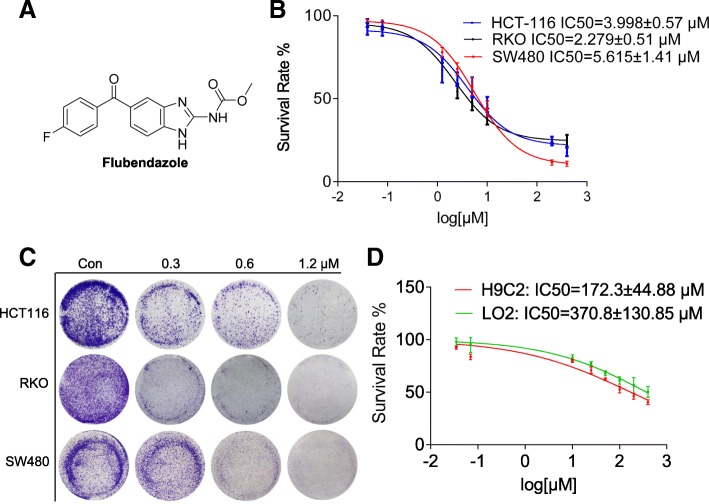
The anthelmintic flubendazole shows promising efficacy to suppress CRC cell proliferation.(**a**) The chemical structure of flubendazole. (**b**) IC50 values of flubendazole in CRC cells. Three CRC cell lines were seeded in 96-well plate and treated with various concentrations of flubendazole (0–400 μM). Proliferation was measured after 48 h of treatment by the MTT assay. The proliferation curves were plotted using the GraphPad Prism 7.0 software. Data are expressed as the mean ± SD of 3 independent experiments. (**c**) Colony forming assay of the indicated cell lines. Cells were incubated with various concentrations of flubendazole for 24 h. On day 7, colonies were fixed and photographed. Representative images are displayed. (**d**). The IC50 values of flubendazole in normal human cardiomyocytes H9C2 and liver cell line LO2. Samples were measured in triplicate and experiments were independently repeated three times

The antitumor activity of flubendazole was evaluated by analyzing its inhibitory effects on cell proliferation (Fig. 1a). Our findings revealed that flubendazole effectively reduces the viability of CRC cells (HCT116, RKO and SW480) in a concentration-dependent manner, with an IC50 of 2–5 μM (Fig. 1b). In addition, flubendazole significantly decreased colony formation in a dose-dependent manner (Fig. 1c), which is quite consistent with the results of the above experiments. The inhibitory effect of flubendazole against normal human cells LO2 and H9C2 was significantly limited compared with cancer cells under the same situation of exposure time, with the 48 h IC50 > 150 μM (Fig. 1d). The doubling time of cancer cells is typically < 24 h while the doubling time of normal cells can be several days. Given that flubendazole effects are likely cell cycle specific, the normal cells wouldn’t have had time to divide in 48 h. This means that the proliferation inhibition in normal cells requires higher doses of flubendazole or more time. Together, these data demonstrated that flubendazole clearly inhibits CRC cells proliferation in a concentration dependent manner, but correspondingly has weak inhibitory effect to normal human cells at the concentrations used in our study. Accordingly, the treatment with flubendazole appears to be partly safety to normal cells and powerfully cytotoxic to CRC cells.

### Flubendazole exhibits pharmacological antitumor activity by blocking STAT3 signaling

**Fig. 2:**
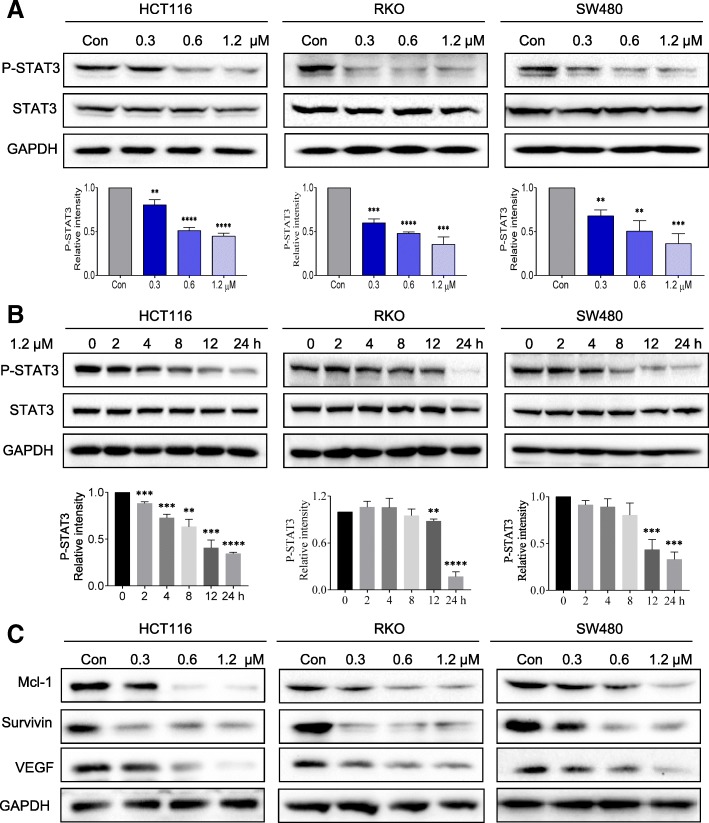
Flubendazole inhibits STAT3 signaling pathway in a dose and time-dependent manner.(**a**) Cells were incubated with the flubendazole at different concentrations, as indicated, for 24 h, the cell lysates were prepared for western blot analysis to determine protein expression of P-STAT3, STAT3 and GAPDH, which was used as a loading control. The results are representative of three replicate experiments**.** (**b**) Flubendazole (1.2 μM) inhibits P-STAT3 in a time-dependent manner. (**c**) Cells were incubated with flubendazole for 24 h. Protein expression was determined by immunoblot analysis. GAPDH was used as a loading control. Results are representative of three independent experiments (**P < 0.01, ***P < 0.001, ****P < 0.0001)

To determine the specific signaling pathway that is involved in the suppression of tumor cell growth, we performed western blot analysis in three CRC cell lines. Treatment with flubendazole caused no obvious change in total STAT3 expression, but it strongly reduced the expression of phosphorylated STAT3 (P-STAT3) in a dose and time-dependent manner (Fig. 2a, b). As the protein expression level and biological effect of the anti-apoptotic factors MCL1 and survivin (*BIRC5*) have been associated with phosphorylation of STAT3 in tumor cells [9, 10], we examined the protein expression level of STAT3 target genes, including MCL1 and survivin. As anticipated, we found that flubendazole decreased expression of MCL1 and survivin in a dose-dependent manner (Fig. 2C and Additional file 1: Figure S1A). STAT3 activation also regulates the expression of VEGF and human cancer angiogenesis and metastasis [10]. We also found the flubendazole significantly downregulated the expression of VEGF (Fig. 2c and Additional file 1: Figure S1B). Furthermore, in order to investigate whether the inhibitory effect of flubendazole on STAT3 is due to the suppression of upstream signaling pathways, we performed western blot analysis to investigate the effect of flubendazole on upstream kinases such as JAK1, JAK2 and SRC. As shown in Additional file 1: Figure S2A, flubendazole inhibited STAT3 phosphorylation partly dependent of the upstream kinases JAK2 and JAK3. Together, these results confirmed that flubendazole suppressed tumor progression by inactivating and/or inhibiting the expression of JAK/STAT3 signaling and STAT3-associated target genes.

### Flubendazole disrupts nuclear translocation of STAT3

**Fig. 3:**
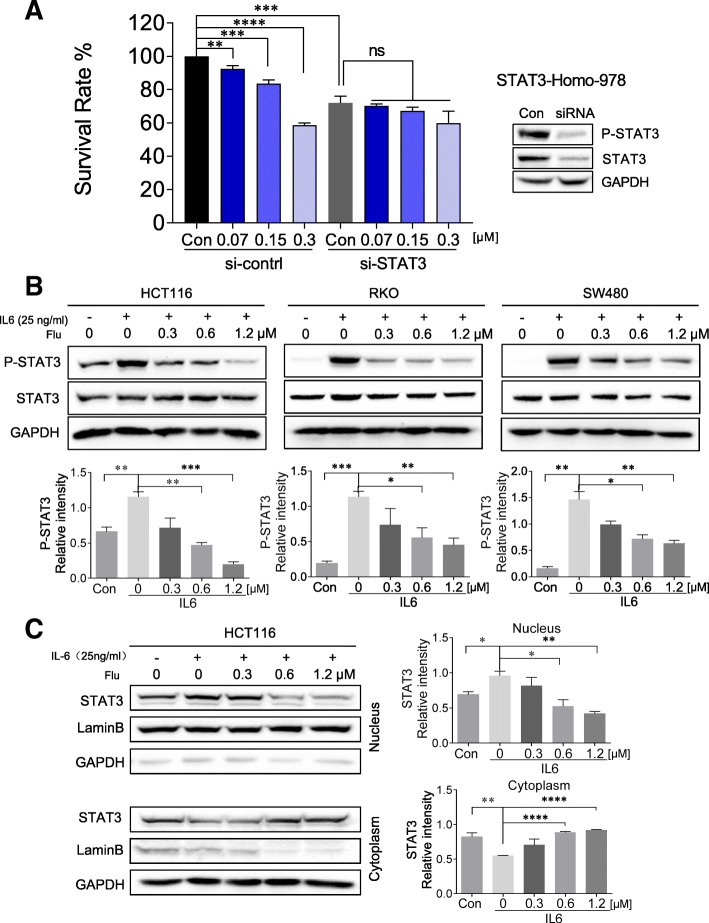
Flubendazole disrupts nuclear translocation of STAT3. (**a**) The siRNA growth experiment measures inhibitory effect of flubendazole on proliferation in HCT116 cells transfected with si-NC or si-STAT3. (**b**) Cells were pretreated with the indicated concentrations of flubendazole (Flu) for 24 h and then stimulated with IL6 (25 ng/mL) for 30 min. Cell extracts were prepared and subjected to the western blot analysis using the indicated antibodies. GAPDH was used as a loading control. Results are representative of three independent experiments. (**c**) HCT116 cells were treated with flubendazole (Flu) for 24 h, and stimulation with IL6 (25 ng/mL) for 30 min before lysing. Protein expression in the cytoplasmic and nuclear extracts was separately determined by immunoblot analysis to determine the cellular distribution of STAT3. Samples were measured in triplicate and experiments were independently repeated three times (*P < 0.05, **P < 0.01, ***P < 0.001, ****P < 0.0001)

Consequently, to further investigated if the growth arrest induced by flubendazole is associated with STAT3 signaling inactivation, we transfected three different sequences of STAT3 siRNAs to down-regulate the expression of STAT3 in HCT116 cells. The results of growth experiment indicated that proliferation inhibition of flubendazole is significantly impaired compared with the cell transfected with negative control siRNA (Fig. 3a and Additional file 1: Figure S2B). It may mean that growth inhibition induced by flubendazole is associated with the suppression of STAT3 in cancer cells. Thus, we next investigated whether the STAT3 activation stimulating by IL6 could be disturbed through the treatment of flubendazole. As shown in Fig. 3b, flubendazole can inhibit IL6-induced STAT3 activation in three CRC cell lines. Moreover, in order to regulate expression of target genes, STAT3 is translocated from the cytosol to the nucleus. Furthermore, IL6-induced STAT3 nuclear translocation was gradually blocked by flubendazole in a concentration dependent manner (Fig. 3c). These results strongly suggest that the antitumor effect of flubendazole is partly mediated through inhibition of the STAT3 signaling pathway.

### Flubendazole activates autophagy in CRC cells

**Fig. 4:**
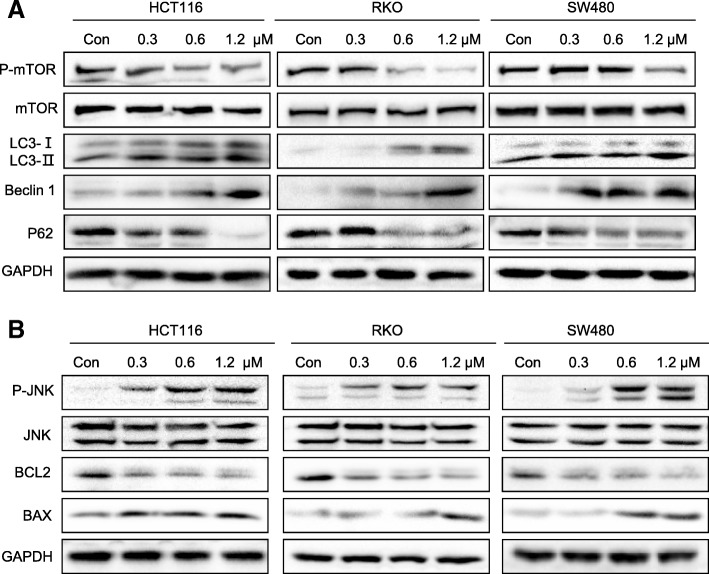
Flubendazole induces cell apoptosis through activation of autophagy.(**a**) Three CRC cell lines were treated with DMSO or flubendazole (0.3, 0.6 and 1.2 μM), lysed and subjected to immunoblotting. (**b**) Cells were incubated with flubendazole for 24 h. Afterwards, western blot analysis of apoptosis-related proteins was performed. GAPDH was used as a loading control. Results are representative of three independent experiments

Flubendazole has been shown to activate autophagy and autophagic flux that may be applicable in disease treatment [13]. We further investigated whether flubendazole exerted similar effects on the progression of tumor growth by activation of autophagy in CRC cell lines. Our data suggested that flubendazole induces autophagy initiation by inactivating mTOR and P62, and upregulating LC3-I/II, which are classical marker of autophagy (Fig. 4a and Additional file 1: Figure S3A). In addition, flubendazole-induced activation of JNK reduced the expression level of BCL2 (Fig. 4b and Additional file 1: Figure S3B). The biological function of Beclin 1 could be limited through binding with BCL2. The inhibition of BCL2 as a result of the blockade of STAT3 and activation of JNK caused the release of Beclin 1 from BCL2-Beclin 1 complexes, which in turn led to the initiation of autophagy (Fig. 4a, b). Importantly, the results shown in Fig. 4b indicated that autophagic initiation and limited expression of BCL2 is involved in cell apoptosis in CRC cells. Collectively, these results suggest that flubendazole induces apoptosis in relation to autophagy activation in CRC cells.

### Flubendazole induces apoptosis in CRC cells

**Fig. 5:**
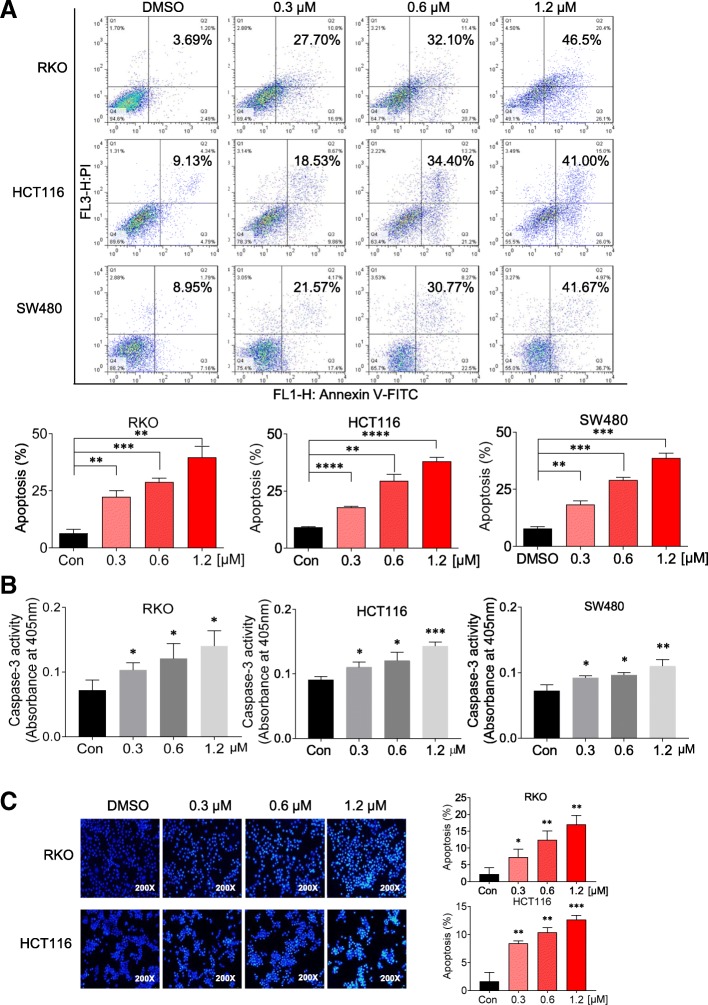
Flubendazole induces tumor cell apoptosis. (**a**) CRC cells were treated with the indicated concentrations of flubendazole and incubated for 48 h. Cells were stained with Annexin V and propidium iodide (PI), and then analyzed by flow cytometry. (**b**) CRC cells were incubated with flubendazole for 36–48 h, caspase 3 activity in the cell extracts was determined with a specific assay kit. (**c**) The apoptotic morphological characteristics of cells and cells stained with Hoechst staining were observed in RKO and HCT116 cells cultured with flubendazole (0, 0.3, 0.6 and 1.2 μM) for 12 h (200×). The images shown are representative of more than three separate experiments (*P < 0.05, **P < 0.01, ***P < 0.001, ****P < 0.0001)

We next assessed the effect of apoptosis in three human CRC cell lines treated with flubendazole at different concentrations (0, 0.3 0.6 and 1.2 μM) for 48 h. As shown in Additional file 1: Fig. 5a, treatment with flubendazole increased the proportion of apoptotic cells in a dose-dependent manner. In addition, using a caspase activity assay, we found that flubendazole dose-dependently effectively increases caspase-3 activity (Fig. 5b). Similar apoptotic effects were indicated by morphological changes in cell nuclei by Hoechst staining (Fig. 5c). Overall, these results suggest that flubendazole significantly promotes apoptosis in CRC cells.

### Flubendazole inhibits growth of CRC tumor xenografts

**Fig. 6:**
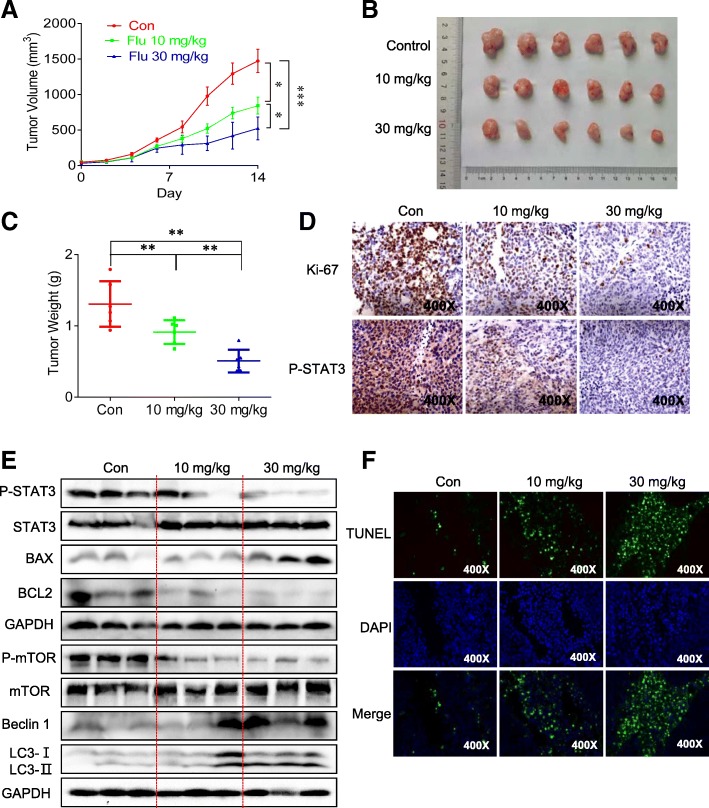
The antitumor activity of flubendazole in vivo. (**a**) Tumor volume was measured every two days. (**b**) The graph of tumors in different groups. (**c**) The weight of the tumors was measured. (**d**) Representative immunohistochemical staining images of cell proliferation marker (Ki-67) and P-STAT3 in tumor tissues. (**e**) The tumor tissues were extracted in lysis buffer, and western blot analysis was performed. (**f**) TUNEL staining results of the tumor tissues (400×), (*P < 0.05, **P < 0.01, ***P < 0.001)

Based on the compelling in vitro evidence, we next assessed the therapeutic efficacy of flubendazole in HCT116 xenograft models. When tumors were visible, mice were administered flubendazole at 10 mg/kg and 30 mg/kg every other day. As shown in Fig. 6a and b, the tumor volume in the mice of the groups treated with flubendazole was markedly reduced compared with that in mice of the control group. Additionally, similar results were obtained with respect to tumor weight (Fig. 6c). Furthermore, there was no clear evidence of cytotoxic activity as measured by the total body weight of the mice and hematoxylin and eosin (H&E) staining (Additional file 1: Figure S4A and S4B). Further research on the mechanism of action of flubendazole on tumor growth was conducted by analyzing tumor protein expression by immunoblot analysis, immunohistochemistry and Tunel assay. These results confirmed that flubendazole significantly reduces the protein level of P-STAT3, promotes autophagy and induces apoptosis in vivo*,* which is good agreement with the in vitro results (Fig. 6d-f). Together, these studies indicate that flubendazole is a promising anticancer agent which effectively inhibits tumor growth by inhibiting the STAT3 signaling pathway and activating autophagy.

### Flubendazole exerts synergistic effect with 5-fluorouracil-based neoadjuvant chemotherapy in CRC

**Fig. 7:**
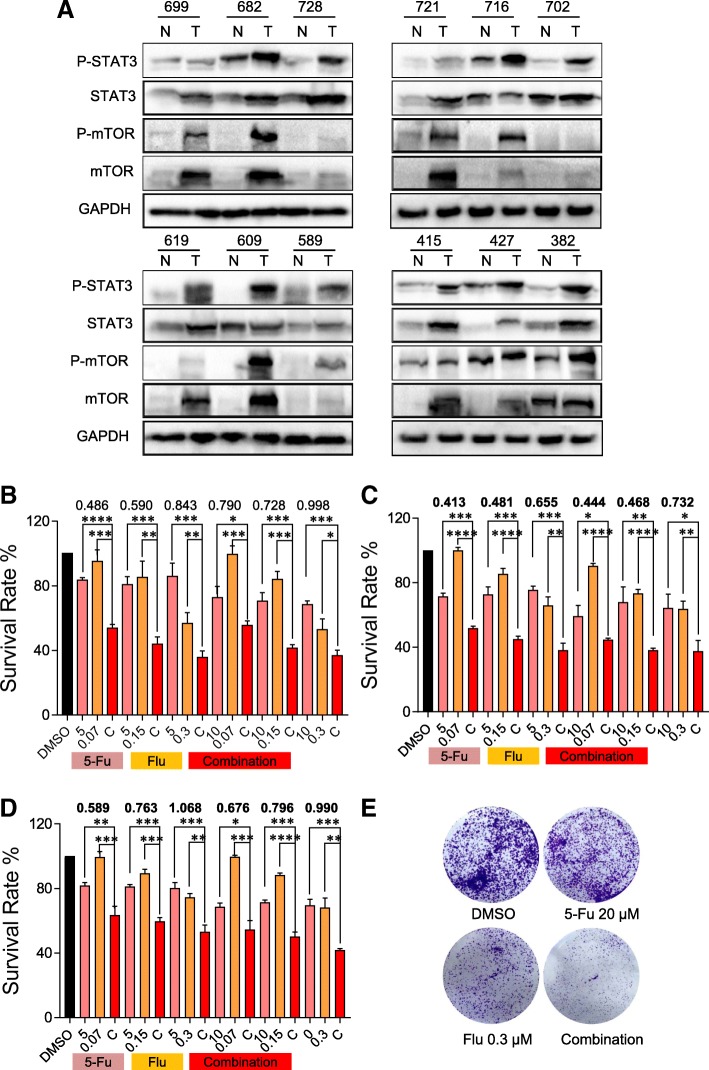
Up-regulation of STAT3 and MTOR expression in CRC tissues and synergism of flubendazole with 5-fluorouracil. (**a**) The protein expression of STAT3 and mTOR in each of the paired CRC tissue samples (T) and adjacent normal tissue samples (N) obtained from the same patients. (**b**) HCT116 cells were treated with serial dilutions of flubendazole (Flu), 5-fluorouracil (5-Fu) or the combination of flubendazole plus 5-fluorouracil. Cell viability was measured after 48 h of treatment by the MTT assay. Survival rates were plotted using the GraphPad Prism software. CompySyn was used for determining the combination index. (**c**) The RKO cells were treated with serial dilutions of flubendazole, 5-fluorouracil or the combination. (**d**) The SW480 cells were treated with serial dilutions of flubendazole, 5-fluorouracil or the combination. (**e**) Representative images of the colony-formation assay with DMSO, 5-fluorouracil (20 μM), flubendazole (0.3 μM), or the combination are presented in HCT116 cell, (*P < 0.05, **P < 0.01, ***P < 0.001, ****P < 0.0001)

Overexpression of STAT3 has been shown plays a role in maintaining tumor progression and low survival rate in various malignancies [21]. However, whether autophagy is associated with the progression of CRC remains unclear. Furthermore, we analyzed the protein expression of STAT3 and mTOR in 12 pairs of CRC tumor and matched adjacent normal tissues by western blot analysis. The results revealed significant upregulation of the expression of STAT3 and mTOR in the tumor tissues compared with the corresponding normal tissue (Fig. 7a). This means that STAT3 or mTOR may be an effective therapeutic target. Accordingly, to further investigate the potential clinical application of flubendazole, we next examined the combination of flubendazole and the chemotherapeutic agent 5-fluorouracil, which is widely used in the neoadjuvant chemotherapy of CRC. As shown in Fig. 7b-d, the combination treatment decreased cell viability much more robustly than either agent alone, with almost all of combination index CI < 0.9. In addition, similar effects were observed in the colony-formation assay. HCT116 cells shown a limited ability to form colonies following the combination treatment. (Fig. 7e). Together, these data revealed that flubendazole may have potential application in inhibiting the cancer cell viability and limiting cancer cell proliferation through its synergism with 5-fluorouracil.

## Discussion

Consideration in terms of security and development costs, the exploration of novel anticancer agent by searching for existing drug repurposing has gradually emerged as a significant direction in preclinical research [14, 22, 23]. In this study, we found that the anthelmintic drug flubendazole could be repurposed as a potential anticancer drug. Our data shown that flubendazole clearly inhibits cell proliferation and induces apoptosis in CRC cells, while in normal human cells it only exhibits weak inhibition (Fig. 1). Additionally, we also revealed that flubendazole effectively blocks the growth of CRC tumor xenografts. Mechanistically, flubendazole exerted its antitumor activity by inhibiting STAT3 and thereby downregulating the expression of its key target genes, including BCL2, MCL1, survivin and VEGF. In addition, it was also determined that flubendazole prevents STAT3 translocation to the nucleus. Moreover, treatment with flubendazole disrupted STAT3 activation leading to cells apoptosis and activation of caspase-3 in CRC cells.

Recently studies have shown that flubendazole can inactivate mTOR signaling by displacing mTOR from lysosome and ultimately promote the maturation of autophagosome and autolysosome [13]. The specific pharmacological action of flubendazole may be associated with disruption of microtubule function [16]. Furthermore, treatment with flubendazole has been found to increase phosphorylation of JNK1 thereby decreasing the expression of BCL2 in BCL2-Beclin 1 complexes through acetylation of microtubules. Accordingly, flubendazole upregulates the release of Beclin 1 and initiation of autophagy. Additional studies indicate that flubendazole activates autophagy through the release of Beclin 1 from BCL2-Beclin 1 complexes and inactivating mTOR [13]. Recent reports further indicate that different subcellular localization patterns of STAT3 affect autophagy in various ways [24]. These recently studies of flubendazole suggest that it may be applicable in cancer treatment. Our results suggested that flubendazole may modulate expression of the STAT3 target gene BCL2 and lead to the initiation of autophagy and cell apoptosis. This represents a completely new idea in the preclinical research of anticancer therapy. In addition, Flubendazole can inhibit microtubule function and displays preclinical activity in leukemia and myeloma [19]. Thus, these data indicated that STAT3 may not be the only target of flubendazole. Potential anti-cancer mechanisms of flubendazole may be more complicated.

We also found that the expression of STAT3 and mTOR was increasing in clinical samples of CRC tissues at the protein levels. Thus, these results suggest the potential application of STAT3 or mTOR as a prognostic and survival indicator in CRC patients. 5-fluorouracil is considered the classic chemotherapy strategy in the treatment of CRC, however, it can cause side effects [7, 25]. Combination therapy may enhance the curative effects and reduce the therapeutic concentration of chemotherapeutic drugs [26]. Notably, we have demonstrated that administration of flubendazole and 5-fluorouracil results in synergistic antiproliferative effects in vitro. Flubendazole has a good inhibitory effect on CRC and can be used in combination with 5-fluorouracil to inhibit CRC. It may enhance the therapeutic effects and reduce the effective concentration. Thus, the precise mechanism-based rationale for the combination therapy will be a significant direction of our future research. Due to the safety and these preclinical studies of flubendazole, clinical trials may consider the application of flubendazole alone or in combination with chemotherapeutic drugs for the treatment of cancer, especially CRC.

## Conclusions

**Fig. 8:**
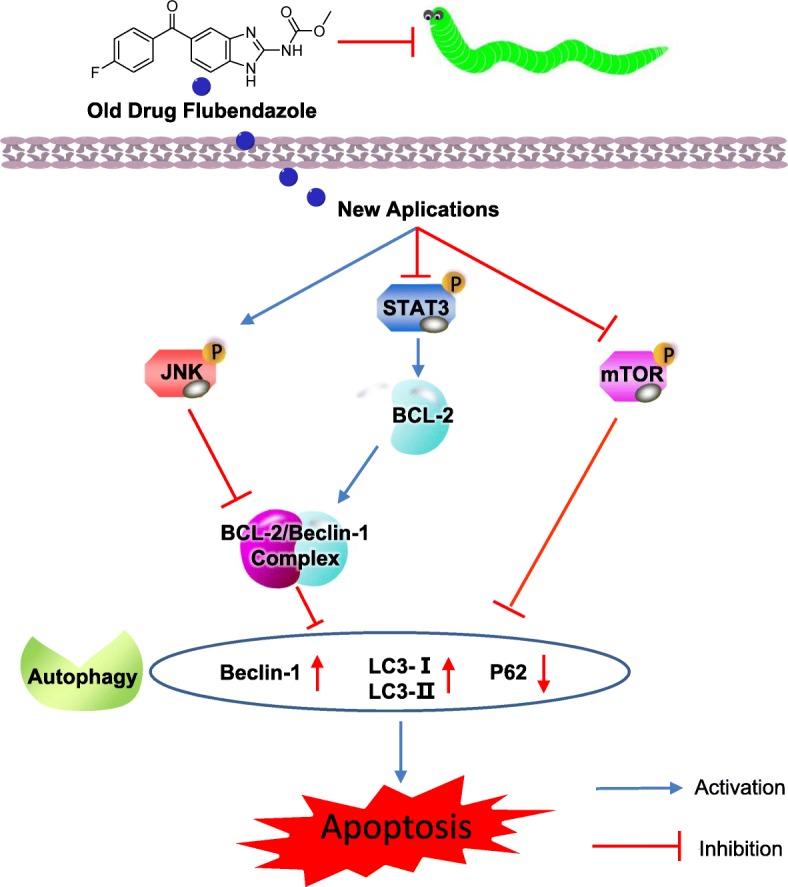
Schematic representation of the potential antitumor mechanism of flubendazole via inhibiting STAT3 and activating autophagy in cancer cells

In summary, development of new drugs still presents enormous challenges and exciting opportunity. No STAT3 inhibitor has yet been approved for clinical application [27]. With the aim of improving both the efficacy and safety of STAT3 inhibitors, through a drug repurposing approach we evaluated the effects of the FDA approved anthelmintic drug flubendazole on CRC and found that it significantly inhibits CRC cell growth in vitro and in vivo. Thus, flubendazole is a potential drug candidate for CRC and other cancers, which acts by inhibiting STAT3 signaling and activating autophagy (Fig. 8). The synergism of flubendazole and 5-fluorouracil represents a new alternative in the field of chemotherapy.

## Additional files

Additional file 1: **Figure S1.** The quantification of intensity of the western blot data about STAT3 target genes. (A) The quantification of intensity about MCL-1 and survivin. (B) The quantification of intensity about VEGF. **Figure S2.** Flubendazole inhibits JAK signaling pathway in a dose-dependent manner. (A) Cells were incubated with flubendazole for 24 h. Protein expression was determined by immunoblot analysis. (B) The siRNA growth experiment measured inhibitory effect of flubendazole on proliferation in HCT116 cells transfected with si-NC or other sequences of si-STAT3.**Figure S3.** The quantification of intensity in protein level of autophagy and apoptosis. (A) The quantification of intensity of proteins which are associated with autophagy. (B) The quantification of intensity of apoptosis-related protein and P-JNK.**Figure S4.** The cytotoxic activity of flubendazole in vivo. (A) Body weight of nude mice was measured once two days. (B) The H&E staining of the major organs (Heart, Liver, Kidney and Lung). (DOCX 386 kb)

Additional file 2: The short tandem repeat (STR) DNA profiles for HCT116. (PDF 316 kb)

Additional file 3: The short tandem repeat (STR) DNA profiles for SW480. (PDF 295 kb)

Additional file 4: The short tandem repeat (STR) DNA profiles for RKO. (PDF 283 kb)

## Data Availability

The datasets used and/or analyzed during the current study are available from the corresponding author on reasonable request.

## Abbreviations

BAX: BCL2-associated X protein
BCL2: B-cell lymphoma 2
BIRC5: Baculoviral IAP Repeat Containing 5 (Survivin)
CI: Combination index
CRC: Colorectal cancer
DMEM: Dulbecco’s Modified Eagle Medium
DMSO: Dimethyl sulfoxide
FBS: Fetal bovine serum
FDA: Food and Drug Administration
GAPDH: Glyceraldehyde 3-phosphate dehydrogenase
HRP: Horseradish peroxidase
IC_50_: The half maximal inhibitory concentrations
IL6: Interleukin 6
JNK: c-Jun N-terminal kinase
LC3: microtubule-associated proteins light chain 3
MCL1: myeloid cell leukemia sequence 1
mTOR: mechanistic target of rapamycin kinase
MTT: Methylthiazolyldiphenyl-tetrazolium bromide
OD: Optical density
P62: Ubiquitin-binding protein p62
PBS: Phosphate-buffered saline
PI: Propidium Iodide
PVDF: Polyvinylidene Fluoride
RPMI: Roswell Park Memorial Institute
SD: Standard deviation
SDS-PAGE: Sodium dodecyl sulfate-polyacrylamidegel
SEM: Standard error of mean
siRNA: Small interfering RNA
STAT3: Signal transducer and activator of transcription 3
TBST: Tris-Buffered Saline and Tween 20
VEGF: Vascular endothelial growth factor
