# Supplementary material for: Flubendazole demonstrates valid antitumor effects by inhibiting STAT3 and activating autophagy
Source: J Exp Clin Cancer Res. 2019 Jul 8;38:293. doi: 10.1186/s13046-019-1303-z (PMC6615228; doi:10.1186/s13046-019-1303-z)
Supplement: Supplementary file 1 — Figure S1. The quantification of intensity of the western blot data about STAT3 target genes. (A) The quantification of intensity about MCL-1 and survivin. (B) The quantification of intensity about VEGF. Figure S2. Flubendazole inhibits JAK signaling pathway in a dose-dependent manner. (A) Cells were incubated with flubendazole for 24 h. Protein expression was determined by immunoblot analysis. (B) The siRNA growth experiment measured inhibitory effect of flubendazole on proliferation in HCT116 cells transfected with si-NC or other sequences of si-STAT3.Figure S3. The quantification of intensity in protein level of autophagy and apoptosis. (A) The quantification of intensity of proteins which are associated with autophagy. (B) The quantification of intensity of apoptosis-related protein and P-JNK.Figure S4. The cytotoxic activity of flubendazole in vivo. (A) Body weight of nude mice was measured once two days. (B) The H&E staining of the major organs (Heart, Liver, Kidney and Lung). (DOCX 386 kb) [file 13046_2019_1303_MOESM1_ESM.docx]

Fig. S1. The quantification of intensity of the western blot data about STAT3 target genes. (A) The quantification of intensity about MCL-1 and survivin. (B) The quantification of intensity

about VEGF.

Fig. S2. Flubendazole inhibits JAK signaling pathway in a dose-dependent manner. (A) Cells were incubated with flubendazole for 24 h. Protein expression was determined by

immunoblot analysis. (B) The siRNA growth experiment measured inhibitory effect of

flubendazole on proliferation in HCT116 cells transfected with si-NC or other sequences of

si-STAT3.

Fig. S3. The quantification of intensity in protein level of autophagy and apoptosis. (A) The quantification of intensity of proteins which are associated with autophagy. (B) The

quantification of intensity of apoptosis-related protein and P-JNK.

Fig. S4. The cytotoxic activity of flubendazole in vivo.(A) Body weight of nude mice was measured once two days. (B) The H&E staining of the major

organs (Heart, Liver, Kidney and Lung).
