# Supplementary material for: Flubendazole demonstrates valid antitumor effects by inhibiting STAT3 and activating autophagy
Source: J Exp Clin Cancer Res. 2019 Jul 8;38:293. doi: 10.1186/s13046-019-1303-z (PMC6615228; doi:10.1186/s13046-019-1303-z)
Supplement: Supplementary file 3 — The short tandem repeat (STR) DNA profiles for SW480. (PDF 295 kb) [file 13046_2019_1303_MOESM3_ESM.pdf]

欣唯诺——唯诺是金

来样单位：温州医科大学附属第一医院

委托项目：细胞鉴定

报告日期：2019-6-19

北京欣唯诺生物科技有限公司　电话：010-57191871
地址：北京市昌平区沙河地铁站东青年创业大厦 B126

细胞 STR 检验报告

委托方：

委托日期：2019 年 6 月 12 日

鉴定日期：2019 年 6 月 19 日

一、　检材：

2019 年 6 月 13 日收到委托人 1 株细胞悬液：管上标记为, SW480。

要求鉴定该株细胞是否为单一来源细胞系, 是否存在交叉污染现象。

二、　检材处理和检验方法：

取适量检材用 chelex 法 提取 DNA，采用 Goldeneye™ DNA ID System 20A 扩增 19 个 STR 位点和性别鉴定位点，使用 ABI 3730xl 型遗传分析仪进行 PCR 产物检测，使用 GeneMapper3.2 软件(Applied Biosystems)对检测结果进行分析，并与 ATCC 和 DSMZ 数据库进行比对。

三、　检验结果：

实验中阴性及阳性对照结果均正确。

细胞株的 STR 位点和 Amelogenin 位点的基因分型结果见附表 1，分型图谱见附图。比对结果见附表 2。

四、　分析说明：

该株细胞 DNA 扩增后图谱清晰，分型结果良好。

五、　检验结论：

SW480 ①该株细胞 DNA STR 分型结果显示，在各基因座均未出现三等位基因现象。细胞中没有发现人类细胞交叉污染。②该细胞与 ATCC 中 SW480Colon AdenocarcinomaHuman 细胞的 STR 数据匹配率为最高，为 100%。推测该细胞即为 SW480Colon AdenocarcinomaHuman。③该株细胞 DNA 分型在 DSMZ 细胞库中找到匹配度 EV 值最高为 1.00 的 2 种细胞，细胞名称分别为 SW-480 和 SW-598。

（此结果仅对本次检材负责）

操作人：杨丽丽　　　　审核人：宋欣欣

备注：

1. 根据 ANSI 制定的国际标准，细胞系的匹配度≥80% 时，认为它们具有相关性，即衍生于共同的祖先细胞；匹配度在 55% 至 80% 之间，需要进一步验证相关性；小于 55%，表明两者不具有相关性。
2. 有效峰为真实的 PCR 条带;小峰和非特异性条带在计算中忽略不计。

本实验依照 Goldeneye^{TM}20 ID System STR 试剂盒提供的实验手段和分析方法进行检测，结果仅供参考。

Standards for Cell Line Authentications

To standardize STR analysis for human cell line authentication, the American Tissue Culture Collection (ATCC) Standards Development Organization Workgroup published ASN-0002-2011, which recommends the use of at least eight STR loci (TH01, TPOX, vWA, CSF1PO, D16S539, D7S820, D13S317 and D5S818) plus Amelogenin for gender identification for human cell line authentication.

附表 1：1 株细胞的 STR 位点和 Amelogenin 位点的基因分型结果

细胞 SW480（图片编号 SW480）		
Marker	Allele 1	Allele 2
D19S433	13	13
D5S818	13	13
D21S11	30	30.2
D18S51	13	13
D6S1043	11	12
D3S1358	15	15
D13S317	12	12
D7S820	8	8
D16S539	13	13
CSF1PO	13	14
Penta D	9	15
AMEL	X	X
vWA	16	16
D8S1179	13	13
TPOX	11	11
Penta E	10	10
TH01	8	8
D12S391	17	17
D2S1338	17	24
FGA	24	24

附图：1 株细胞的 STR 分型图谱

SW480

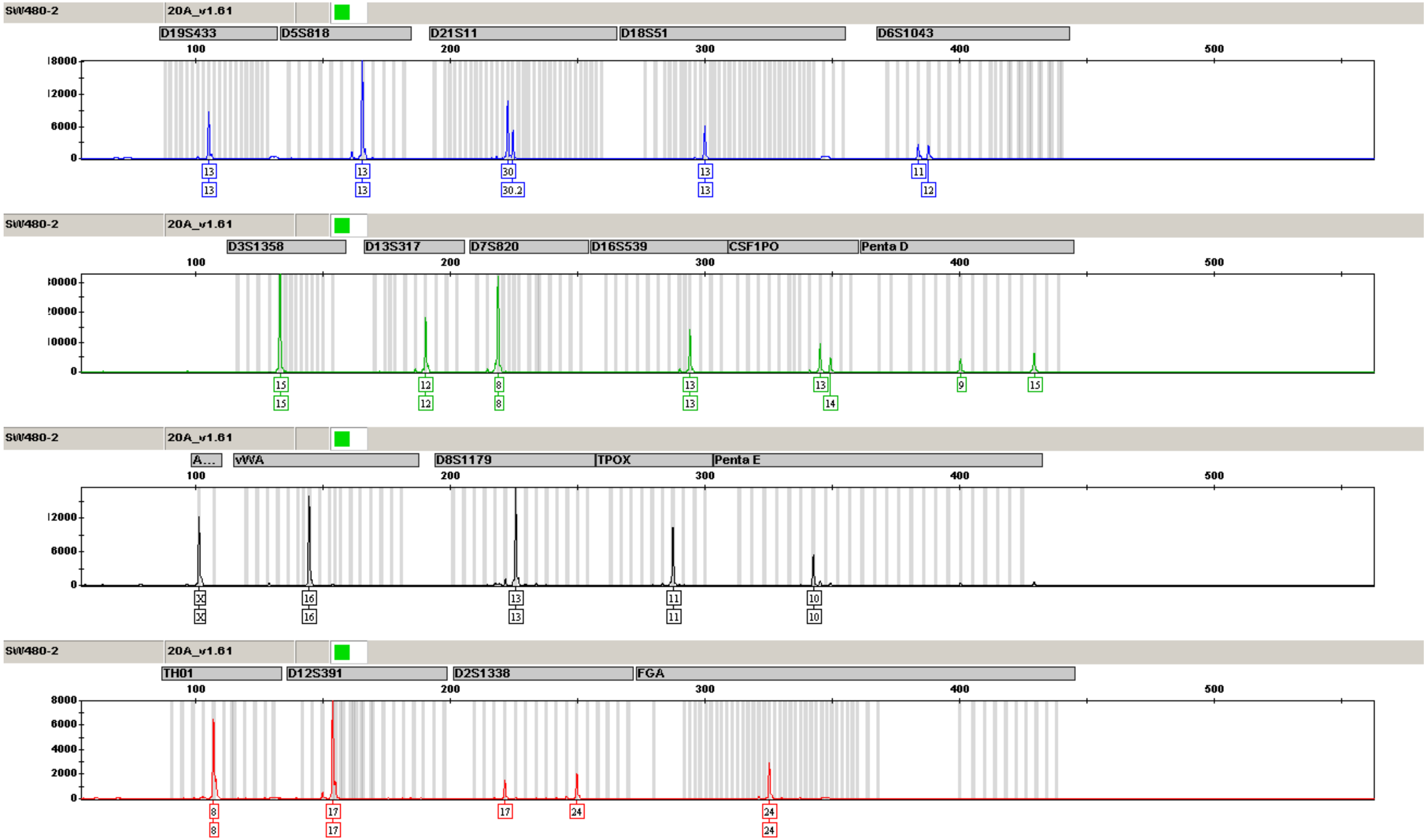

北京欣唯诺生物科技有限公司　电话：010-57191871

地址：北京市昌平区沙河地铁站东青年创业大厦 B126

附表 2：细胞分型比对结果

细胞 SW480 分型 ATCC 比对结果

% Match	Sample Count	Matches	Atcc Number	Designation	D5S818	D13S317	D7S820	D16S539	vWA	TH01	AMEL	TPOX	CSF1PO
			鉴定细胞株	SW480	13, 13	12, 12	8, 8	13, 13	16, 16	8, 8	x, x	11, 11	13, 14
100	10	10	CCL-228	SW480Colon AdenocarcinomaHuman	13	12	8	13	16	8	X	11	13, 14
91	11	10	CRL-7940	SW527Mammary GlandHuman	13	12	8	9, 13	16	8	X	11	13, 14
83	12	10	CCL-227	SW620Colon AdenocarcinomaHuman	13	12	8, 9	9, 13	16	8	X	11	13, 14

细胞 SW480 分型 dsmz 比对结果

**EV**	**Cell No.**	**Cell name**	**Locus names**								
			**D5S818**	**D13S317**	**D7S820**	**D16S539**	**VWA**	**TH01**	**AM**	**TPOX**	**CSF1PO**
	Query (SW480)		***13,13***	***12,12***	***8,8***	***13,13***	***16,16***	***8,8***	***x,x***	***11,11***	***13,14***
1.00(36/36)	313	SW-480	**13,13**	**12,12**	**8,8**	**13,13**	**16,16**	**8,8**	**X,X**	**11,11**	**13,14**
1.00(36/36)	CCL-228	SW480 [SW-480]	**13,13**	**12,12**	**8,8**	**13,13**	**16,16**	**8,8**	**X,X**	**11,11**	**13,14**
1.00(36/36)	CRL-2176	SW-598	**13,13**	**12,12**	**8,8**	**13,13**	**16,16**	**8,8**	**X,X**	**11,11**	**13,14**
0.94(34/36)	CRL-7940	SW 527	**13,13**	**12,12**	**8,8**	9,**13**	**16,16**	**8,8**	**X,X**	**11,11**	**13,14**
0.89(32/36)	CCL-227	SW620 [SW-620]	**13,13**	**12,12**	**8,**9	9,**13**	**16,16**	**8,8**	**X,X**	**11,11**	**13,14**
